# A dissociation between stopping and switching actions following a lesion of the pre-supplementary motor area

**DOI:** 10.1016/j.cortex.2014.08.004

**Published:** 2015-02

**Authors:** R. Edward Roberts, Masud Husain

**Affiliations:** a Academic Department of Neuro-otology, Division of Brain Sciences, Imperial College London, London, UK; b Nuffield Department of Clinical Neurosciences, University of Oxford, Oxford, UK; c Department of Experimental Psychology, University of Oxford, Oxford, UK

**Keywords:** Pre-SMA, Cognitive control, Executive function, Frontal

## Abstract

**Introduction:**

Although the pre-supplementary motor area (pre-SMA) is one of the most frequently reported areas of activation in functional imaging studies, the role of this brain region in cognition is still a matter of intense debate. Here we present a patient with a focal lesion of caudal pre-SMA who displays a selective deficit in updating a response plan to switch actions, but shows no impairment when required to withhold a response – stopping.

**Materials & methods:**

The patient and a control group underwent three tasks designed to measure different aspects of cognitive control and executive function.

**Results:**

The pre-SMA patient displayed no impairment when responding in the face of distracting stimuli (Eriksen flanker paradigm), or when required to halt an on-going response (STOP task). However, a specific deficit was observed when she was required to rapidly switch between response plans (CHANGE task).

**Conclusions:**

These findings suggest that the caudal pre-SMA may have a particularly important role in a network of brain regions required for rapidly updating and implementing response plans. The lack of any significant impairment on other measures of cognitive control suggests that this is not likely due to a global deficit in cognitive control. We discuss the implications of these results in the context of current theories of pre-SMA function.

## 1 Introduction

The pre-supplementary motor area (pre-SMA) in humans is located in the dorsomedial frontal cortex, rostral to the supplementary motor area (SMA) and dorsal to the cingulate motor areas (Nachev, Kennard, & Husain, 2008). Although the pre-SMA is the most frequently activated brain region in neuroimaging studies (Behrens, Fox, Laird, & Smith, 2012), there is still no consensus on its function. In terms of its connectivity with other brain regions, pre-SMA displays a profile that is quite distinct to neighbouring SMA, with more of its connections projecting to dorsolateral prefrontal cortex than motor areas. This is based on both neuroimaging data in humans (Johansen-Berg et al., 2004; Kim et al., 2010) and animal studies (for a review see Nachev et al., 2008).

Despite the wealth of information from neuroimaging, decoding the precise role of pre-SMA remains to be established and has proven to be challenging, due to its apparent involvement in situations which could imply many different functions (Nachev et al., 2008). In humans the principal focus of a large number of studies has been to identify the contribution of pre-SMA to the performance of tasks designed to measure aspects of cognitive control and executive function (Curtis & D'Esposito, 2003; Nachev, Rees, Parton, Kennard, & Husain, 2005; Shima & Tanji, 2000). These paradigms often require participants to rapidly inhibit or alter a pre-potent response (Curtis & D'Esposito, 2003; Logan & Cowan, 1984; Mostofsky et al., 2003; Nachev et al., 2005), or to respond accurately in the presence of distractors (Botvinick, Nystrom, Fissell, Carter, & Cohen, 1999; Luks, Simpson, Dale, & Hough, 2007; Shima & Tanji, 2000). To date, evidence from functional imaging has implicated pre-SMA in stopping an on-going response (Aron & Poldrack, 2006; Obeso, Robles, Marrón, & Redolar-Ripoll, 2013; Picard & Strick, 1996; Sharp et al., 2010), selecting between conflicting response alternatives (Forstmann, van den Wildenberg, & Ridderinkhof, 2008; Garavan, Ross, Kaufman, & Stein, 2003; Mostofsky & Simmonds, 2008; Nachev et al., 2005; Van Gaal, Scholte, Lamme, Fahrenfort, & Ridderinkhof, 2011), and switching from automatic to voluntary action (Curtis & D'Esposito, 2003; Isoda & Hikosaka, 2007; Nachev, Wydell, O'Neill, Husain, & Kennard, 2007; Ullsperger & von Cramon, 2001).

Diffusion tensor imaging in humans has also been used to describe a triangular structural network linking pre-SMA, inferior frontal cortex (IFC) and subthalamic nucleus (STN) (Aron, Behrens, Smith, Frank, & Poldrack, 2007), which is also thought to exist in non-human primates (Nambu, Takada, Inase, & Tokuno, 1996). It has been proposed that such a network may enable the rapid braking of an initiated action by providing a ‘hyper-direct’ connection from pre-SMA to STN (Aron et al., 2007; Nambu et al., 1996). This structural connection has led to the suggestion that the pre-SMA may play a key role in stopping on-going responses – possibly explaining one facet of pre-SMA function. However, even within the area of cognitive control, it remains unclear precisely what contribution is made by pre-SMA in situations with different response requirements.

Some have proposed that pre-SMA may be a key node in brain networks responsible for the voluntary control of action (Lau, Rogers, Ramnani, & Passingham, 2004; Rushworth, Hadland, Paus, & Sipila, 2002), as volition or self-generated actions (not externally cued) appear to be a common factor across experimental findings. For example, the *Bereitschaftspotential* – a negative premotor potential recorded over central frontal electrodes in humans – has larger peak amplitudes with self-initiated actions (Deecke & Kornhuber, 1978); while in monkeys, lesions of the pre-SMA impair the ability to initiate arbitrary movements to obtain a reward, but the effect is ameliorated if the animals are cued with an external tone (Thaler, Chen, Nixon, Stern, & Passingham, 1995).

Unilateral inactivation of monkey pre-SMA with muscimol has been found to induce deficits in sequence learning, but performance of previously well-learnt sequences was left intact (Nakamura, Sakai, & Hikosaka, 1999). This has led to the suggestion that this might reflect an impairment of the mechanism responsible for updating the association between the correct action given *current* conditions. Therefore, it is possible that deficits in self-initiated action observed after SMA/pre-SMA disruption might arise from a failure to make the appropriate connection between the action to be initiated in a novel situation (Nachev et al., 2008).

Trans-cranial magnetic stimulation (TMS) has also been employed to measure physiological interactions between pre-SMA and other brain regions associated with response selection. This has demonstrated that in the presence of response conflict, pre-SMA facilitates motor-evoked potentials in M1 during action reprogramming (Mars et al., 2009), and suppresses unselected response options (Duque, Olivier, & Rushworth, 2013). TMS over pre-SMA has been associated with an increased delay in the ability to inhibit responses (Cai, George, Verbruggen, Chambers, & Aron, 2012), but there is also evidence that activity in pre-SMA can occur before stopping is initiated, which would be indicative of a role in selecting rather than implementing responses (Swann et al., 2012). However, a caveat of this approach is that TMS stimulation which induces a transient ‘lesion’ may also propagate to other brain networks. Similar effects on network function have also been observed following anatomical focal lesions, dependent on the position of the brain area within the network architecture and degree of white matter involvement (Gratton, Nomura, Pérez, & D'Esposito, 2012).

Although cognitive control, self-initiated action and sequence learning may not be mutually exclusive functions, providing an overarching framework which can account for the range of such complex behaviour has proven difficult. Due to the extremely rare incidence of focal damage to this brain area in humans, only a very small number of lesion studies of pre-SMA have been reported. Moreover, these reports have included patients whose lesions were not entirely constrained within the borders of the pre-SMA, extending into sections of either cingulate gyrus, superior frontal gyrus or SMA (Floden & Stuss, 2006; Nachev et al., 2007). As these adjacent brain areas have also been implicated in cognitive control tasks (particularly anterior cingulate), it is not possible to entirely disambiguate their possible contribution to the deficits observed in these studies. To our knowledge there has been no report of a patient whose lesion is entirely constrained within the borders of the pre-SMA.

Here we present a young patient with a highly focal, unilateral lesion of the caudal pre-SMA. Since pre-SMA has frequently been associated with cognitive control and executive function, we chose to investigate how this might have affected performance on three standard tasks, each of which indexes a different aspect of response selection or inhibition. The STOP-signal task assesses the ability to inhibit an on-going response, whereas the CHANGE-signal task requires the participant to rapidly switch to a different response plan. Finally the Eriksen flanker task measures how quickly an individual is able to select between conflicting response plans that are activated simultaneously. Together these tasks employ similar stimuli with different rules, to explore specific aspects of executive function.

Surprisingly we found that she did not display a significant impairment when asked to stop an action (STOP task), but was significantly impaired when switching between response plans (CHANGE task). The patient also displayed no significant deficit when processing conflict at the level of the stimulus (Eriksen Flanker). Remarkably, it appears that this lesion of the caudal pre-SMA impaired the ability to rapidly switch between overt responses, whilst leaving stopping behaviour intact. We discuss these findings in the context of the current literature and the implications for understanding the role of pre-SMA in voluntary action.

## 2 Materials and methods

### 2.1 Patient participant

Patient KP is a 28-year-old, right-handed woman who was diagnosed with epilepsy, following the onset of simple partial seizures. Following a subsequent grand mal seizure later in the year, further MRI investigations revealed a very small cavernoma (a blood vessel anomaly, also sometimes referred to as a cavernous haemangioma). This was located on the medial aspect of the right superior frontal gyrus. At the time, KP was experiencing complex partial seizures with secondary generalisations, and the cavernoma was subsequently resected.

**Fig. 1:**
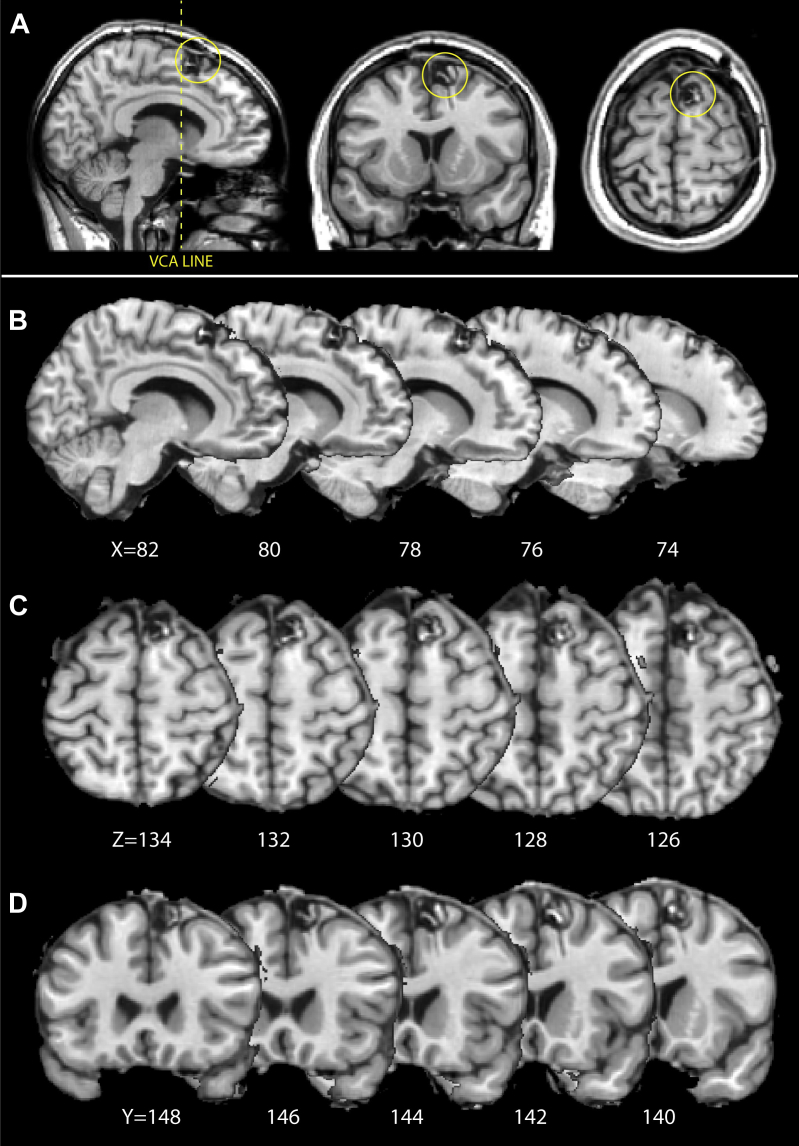
Lesion location. A) A high resolution T1-weighted MRI scan of patient KP in native space, the lesion location is circled in yellow. (B, C, D) The patient's brain image was normalised to standard MNI space and subsections show cross-sections for sagittal, axial and coronal sections, respectively. In panel B the VCA line is marked as a black vertical line, posterior to the lesion location. The lesion clearly lies medial to the superior frontal sulcus, anterior to paracentral sulcus, and dorsal to cingulate sulcus.

A follow-up structural scan 4 months after surgery demonstrates the focal nature of the lesion, which lies medial to the superior frontal sulcus and rostral to the paracentral sulcus. The paracentral sulcus has previously been demonstrated to be a useful landmark for the location of the supplementary eye field (SEF) (Grosbras, Lobel, Van de Moortele, LeBihan, & Berthoz, 1999), which lies at the caudal border of the pre-SMA; thus this lesion lies well within the pre-SMA. The sagittal sections in Fig. 1A and B illustrate that the lesion is clearly located dorsal to the cingulate sulcus (and cingulate motor areas). Importantly, therefore, any behavioural deficit observed in this patient cannot be attributed to direct damage to ACC or SMA as the boundaries of the lesion do not encroach on the surrounding brain areas.

### 2.2 Control participants

A group of 10 healthy volunteers (7 males) were recruited to act as a control group, mean age = 30.9, SE = .63). All participants were right-handed (mean score = 90, SE = 2.6); Edinburgh Handedness test (Oldfield, 1971). All reported normal or corrected-to-normal colour-vision and no subject was taking any medication. Participants were reimbursed £8/h to cover travel expenses.

### 2.3 Assessment

#### 2.3.1 Clinical neuropsychological evaluation

A clinical neuropsychological assessment of KP was conducted before and after surgery (Table 1). The assessment included measures of intellectual function (Verbal IQ, Performance IQ), memory (recognition memory test for words and faces) and focal cognitive abilities (Naming skills, VOSP silhouettes and Cube Analysis, Stroop colour-word, Trails B, Symbol Digit Modalities test).

### 2.4 Experimental tasks

#### 2.4.1 Behavioural tasks

##### 2.4.1.1 STOP task

**Fig. 2:**
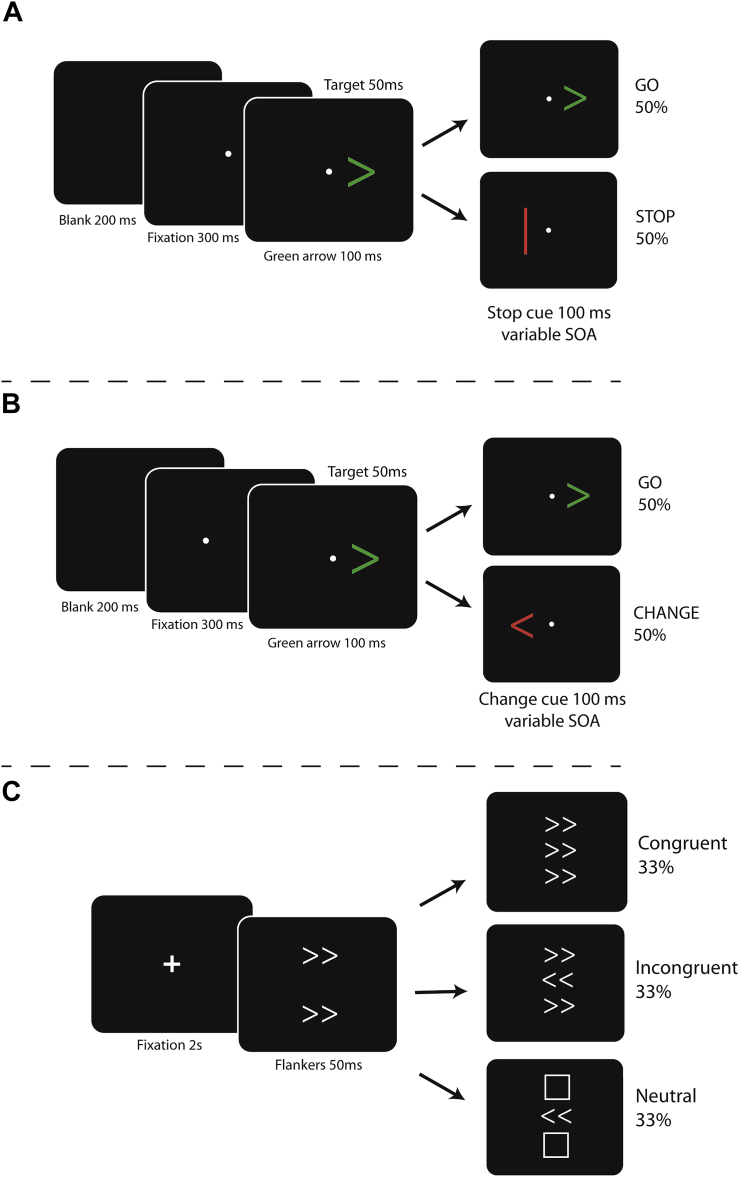
Design of behavioural paradigms. A) The CHANGE of plan task. Participants must respond to the direction of the green Go arrow unless they see the red CHANGE arrow, whereupon they must change their response. B) The STOP-signal task. Participants must respond to the direction of the green Go arrow unless they see the STOP bar, when they must withhold their response. C) Eriksen flanker task. Participants must respond to direction of the central arrow whilst ignoring the peripheral distractors.

In the STOP task (Fig. 2A) participants are instructed to respond as quickly as possible to the direction of an imperative GO stimulus. In this version of the task, which is a variant of a CHANGE task we have presented previously (Roberts, Anderson, & Husain, 2010), the GO signal was a green arrow pointing left or right, and participants were required to press either a left or right response key using the corresponding index finger (Logan, Cowan, & Davis, 1984). On 50% of trials the GO signal was the only stimulus presented. On the remaining trials the GO signal would be followed, after a variable delay, by a STOP signal: a vertical red bar. In the event of a STOP signal, participants were instructed to attempt to withhold their response. They were also instructed to avoid waiting for a STOP signal.

Throughout the course of the experiment the stimulus onset asynchrony between the GO and STOP signals was varied parametrically using a staircase algorithm in response to the performance of the participant (Levitt, 1971). This was in order to determine the delay at which each participant was able to correctly respond to a STOP signal on 50% of trials; the STOP-signal reaction time (SSRT). In order to account for drift in reaction times, a cubic spline was fitted to the CHANGE-signal reaction time (CSRT) data, guided by the shape of Go responses. This method uses the local variation of the Go distribution to interpolate across STOP trial data points. The resulting distribution provides an approximation of the local Go RT for each Stop trial, which is then used to calculate the SSRT.

##### 2.4.1.2 CHANGE task

The CHANGE task (Fig. 2B) employed a similar design to the STOP task. However, instead of a STOP signal, participants were presented with a CHANGE signal – a red arrow pointing in the opposite direction to the GO signal (Roberts et al., 2010). Participants were instructed to respond as quickly as possible to the GO signal, unless they saw a CHANGE signal, in which case they had to attempt to respond with the finger corresponding to the direction of the CHANGE signal instead. The delay between the GO and CHANGE signals was varied in the same manner as described in the STOP task in order to find delay at which each individual was able to change their response on 50% of trials; the CSRT.

##### 2.4.1.3 Eriksen flanker task

In this version of the flanker task (Roberts et al., 2010) participants were asked to respond to the direction of a central target arrow using their index fingers. The target arrow could point either left or right, and presented above and below it were distracting objects (Fig. 2C). These could be either arrows pointing in the same direction as the target (congruent), the opposite direction (incongruent) or squares (neutral). Participants were instructed to respond as quickly and as accurately as possible to the central target arrow, and ignore the distractors. Performance on this task is measured in terms of latency of response to all three stimulus types.

In addition, performance is also measured by comparing the relative differences in reaction time between the three conditions, thus providing three additional indices of.

• **Pure Cost** (incongruent-neutral RT)
• **Benefit** (neutral-congruent RT) and
• **Incongruence Cost** (incongruent-congruent RT).

These measures are often used to estimate the level of positive (facilitating) and negative (interference) effects on reaction time evoked by flankers, with higher incongruence costs usually regarded as indicative of poorer cognitive control on this task. Intra-individual coefficient of variation (ICV) is calculated by dividing the variance in reaction times to neutral stimuli by the mean response (Stuss, Murphy, Binns, & Alexander, 2003). This provides an estimate of the consistency of an individual's responses, and patients with frontal lesions have previously demonstrated impairments on this metric (Stuss et al., 2003).

### 2.5 Procedure

**Fig. 3:**
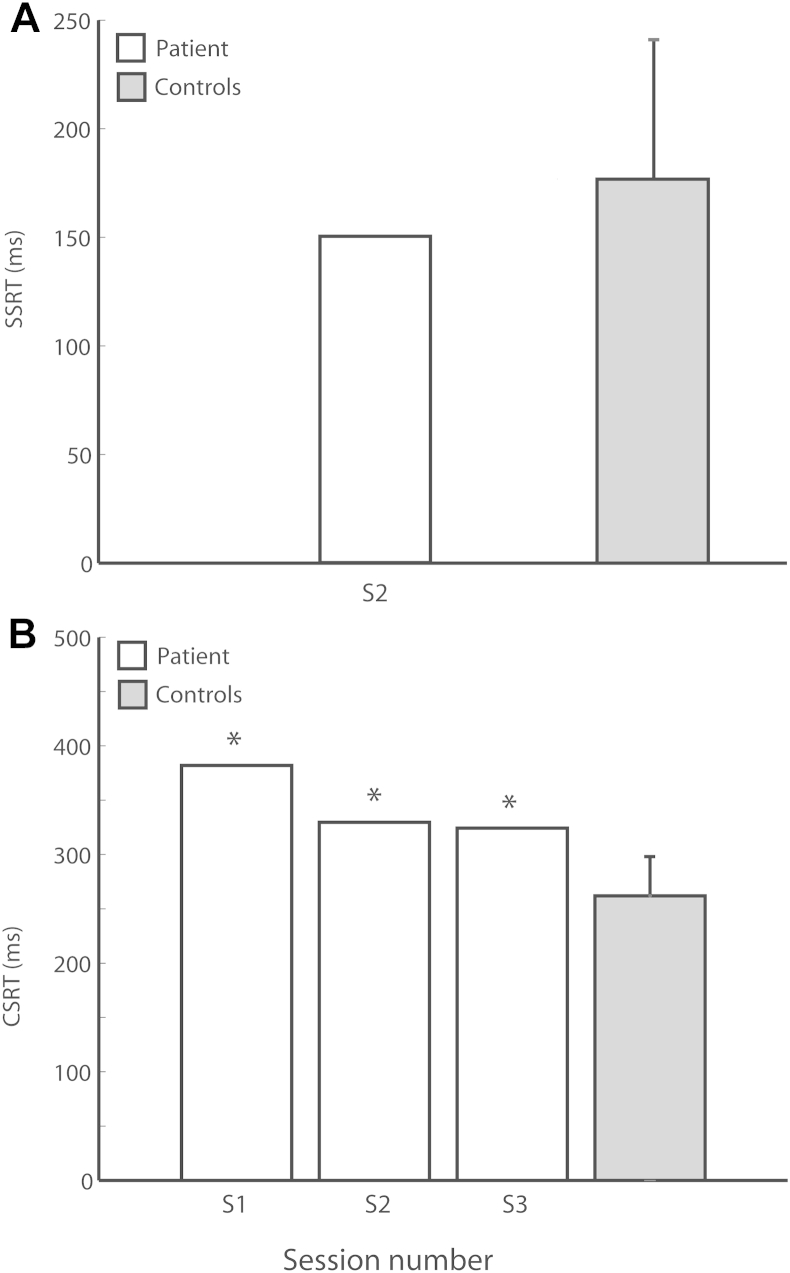
Behavioural findings. Main findings from behaviour experiments. A) STOP task. KP demonstrated no significant difference in performance on this task compared to the control group. B) CHANGE task. KP demonstrated significantly increased latency when required to change responses (CSRT) in all three testing sessions.

All participants were tested in a quiet room with neutral lighting conditions. For the purposes of this experiment, KP was tested on three occasions starting 4 weeks after surgery; see Table 1 for testing protocol. The first session was held 30 days after surgery. The legend of Fig. 3 denotes the session at which the testing took place, labelled S1–S3 (respectively, 4, 10 and 15 weeks post-surgery). Each task took around 30 min to complete, but it was not possible to test KP on CHANGE, STOP and Flanker tasks on all three occasions due to time constraints.

### 2.6 Data analysis

In order to determine whether there was a significant difference between the behaviour of the patient and the control group, confidence limits were employed as described by Crawford and Garthwaite (Crawford & Garthwaite, 2002; Crawford, Garthwaite, & Porter, 2010). This method has become widely used to compare a single case with healthy individuals (Couto et al., 2012). All comparisons are made using a one-tailed level of significance (*p* < .05) because there were explicit predictions about the pattern of results based on the previous neuroimaging literature discussed in the Introduction. The figures presented are shown with ± one standard deviation.

## 3 Results

### 3.1 Clinical and neuropsychological results

KP did not demonstrate any decrements in intellectual function or memory following surgery for her right-hemisphere cavernoma, when tested 15 weeks after surgery (see Table 1). There were no significant changes in focal cognitive ability, except a very mild decline in her performance on the Symbol Digit Modalities Test, on which she was considered borderline impaired, whereas she had previously been average.

### 3.2 Experimental task results

#### 3.2.1 STOP task

KP was tested once on the STOP task, on the second occasion we saw her (Table 1). The SSRT provides an estimate of the time required for an individual to correctly inhibit an initial response on 50% of trials. On this task KP's SSRT (150 msec) was not significantly different (*t* = −.78; *p* > .22) to the control group (mean = 177 msec, SD = 32.1; Fig. 3A).

KP's leftward SSRT was longer than rightwards (12 msec), but this deviation was not significantly different to the controls (*t* = .29; *p* > .39) who also showed slightly greater leftward slowing (7.3 msec, SD = 15.4). In terms of GO reaction time, KP (532 msec) was not significantly different to the control group (mean = 434, SD = 114.3; *t* = .82). She demonstrated virtually no lateralisation in GO reaction time, being only 2 msec quicker when making leftward responses. This was not significantly different to the control group (*t* = −.14; *p* > .45), who overall were slightly slower when making leftward responses (5 msec, SD = 20.9). Thus, KP's performance on the STOP task was entirely within normal limits when assessed (Session 2, S2).

#### 3.2.2 CHANGE task

KP was tested three times on the CHANGE task over the course of 10 weeks (see Table 1). Performance on this paradigm uses a similar metric to the STOP-signal paradigm, however here the CHANGE-signal reaction time (CSRT) reflects the time taken to inhibit an initial response and then correctly execute a second response on 50% of trials.

In the first session (S1), four weeks after surgery, KP's CSRT (382 msec) was significantly higher (*t* = 2.85; *p* < .01) than the control group (mean = 268 msec, SD = 37.7), see Fig. 3B. KP also demonstrated a highly significant lateralisation in CSRT (*t* = 2.6; *p* < .005; paired-samples *t*-test), with leftward CSRT 46 msec slower than rightward. This lateralisation was significantly different to the control group (*t* = 2.61; *p* < .028), who demonstrated a leftward slowing of only 6 msec (SD = 4.6). Both leftward and rightward CSRT measurements were still highly significantly different to the controls (*t* = 3.05; *p* < .007).

Importantly, in terms of GO reaction time KP (mean = 435 msec) was *not* significantly slower than the control group (mean = 395 msec, SD = 160.1; *t* = .24). She did demonstrate an increased latency in responding to leftward GO signals (11 msec), but this was also not significantly different to the controls (*t* = −.17) who showed a similar lateralisation (mean = 14.9 msec, SD = 21.9).

In the second testing session (S2), 10 weeks after surgery, KP's CSRT had reduced to 329 msec. Despite this improvement, KP was still significantly impaired relative to the control group (*t* = 2.2; *p* < .028). In this session KP's GO reaction time had increased (581 msec), but this was not significantly higher than the controls (*t* = .82, *p* > .43). Nor was the lateralisation in her responses significantly different to the controls in this session in terms of Go responses (*t* = 1.04) or CSRT (*t* = −.83).

In the third session (S3), 15 weeks after surgery, KP's CSRT (324 msec) had reduced by a small amount relative to session S1. However, she still remained significantly impaired relative to the controls (*t* = 2.038; *p* < .036). KP's GO reaction time improved in this session (382 msec), and was again not significantly different to the controls (*t* = −.077), neither was her lateralisation in responding in terms of Go reaction time (*t* = .913) or CSRT (*t* = .738).

Thus, KP demonstrated a consistent impairment on the CHANGE task in all three testing sessions, and a lateralised leftward slowing in CSRT in the first session. Note that on the session where we were able to test her on both the STOP and the CHANGE tasks, she performed normally on the former but was impaired on the latter (compare Fig. 3A and B).

#### 3.2.3 Eriksen flanker task

KP's performance on the Eriksen flanker task was assessed in two separate sessions (S2 and S3). In session S2 there were significant differences in reaction time between KP and the controls, but to all three stimulus types. Her reaction time when responding to *congruent* stimuli (468 msec) was significantly longer (*t* = 2.38; *p* < .021) than the control group (mean = 383.7 msec, SD = 34.1). Similarly when responding to neutral stimuli (502 msec *vs* controls mean = 408 msec, SD = 34.4; *t* = 2.56; *p* < .016). The most significant difference between KP's reaction time (570 msec) and the control group was in response to incongruent stimuli where there was a 112 msec increase in latency relative to the control group (458 msec, SD = 35.0; *t* = 3.14; *p* < .001). Thus, in session S2, KP showed overall slowing across all conditions.

In terms of lateralisation of response, KP demonstrated significant leftward slowing compared to rightward responses (*t* = 2.1; *p* < .02; paired-samples *t*-test) on congruent and neutral trials; but no significant difference in response to incongruent stimuli. However, these differences between leftward and rightward movements were not significantly different to the control group on congruent (KP = 20.4 msec; Controls = 10 msec, SD = 18.0), incongruent (KP = −3.2 msec; Controls 16 msec, SD = 19.3), or neutral stimuli (KP = 24.5 msec; Controls = 21 msec, SD = 15.5).

We also calculated the relative differences in reaction time between the stimuli to assess whether KP was more susceptible to interference effects than the controls. KP's reaction time **Benefit** (34 msec) was not significantly different (*t* = 1.57) to the control group (mean = 24.9 msec, SD = 6.6). However, her **Pure Cost** (68 msec) was significantly higher (*t* = 3.97; *p* < .001) than the controls (mean = 49.8 msec, SD = 4.06). In addition, there was also a significant difference between the **Incongruence Cost** measures where KP (102 msec) demonstrated a 27 msec increased latency compared to the control group (mean = 75 msec, SD = 8.08; *t* = 3.35; *p* < .001). KP's accuracy in responding (97%) was not significantly different to the control group (mean = 94.2%, SD = 5; *t* = .56). We also calculated KP's ICV (4.49), but this was again not significantly different to the controls (mean = 3.98, SD = .89; *t* = .539).

It is possible that the large increase in incongruence costs demonstrated by KP in session 2 could have been a product of generalised slowing, rather than a specific impairment when responding to incongruent stimuli. To investigate this possibility, the *ratio* between neutral reaction time and the three incongruence measures was examined. If KP were to demonstrate a significant deviation from the controls on these measures, this might be evidence that her incongruence costs were not just a product of increased reaction times. The analysis demonstrated that the ratio of neutral reaction time to **Incongruence Cost** (KP = .21; Controls = .18, SE = .022), **Pure Cost** (KP = .14; Controls = .12, SE = .014) or **Benefit** (KP = .068; Controls = .059, SE = .015) there was no significant difference between KP and the control group. Therefore it is likely that KP's higher incongruence costs in the first session were simply a consequence of a general increased latency in responding following her lesion.

In the following session (S3) KP's reaction times improved and there was now no significant difference between her reaction time to congruent (422 msec), incongruent (495 msec) or neutral stimuli (440 msec), compared to the control group. Nor were there any significant differences between any of the incongruence measures and the controls. In this session KP again demonstrated no significant differences in accuracy (94%) to the control group, and her consistency (ICV) in responding to neutral stimuli increased relative to the previous session (4.91), but was not significantly higher than in the control group (mean = 3.98, SD = .89; *t* = .99).

In summary, in the first session using the flanker task (S2), KP was consistently slower in responding to all three types of stimuli. KP also demonstrated significantly larger incongruence costs, but this is likely a product of generalised slowing. In the second Flanker session (S3), KP demonstrated no significant impairment compared to controls.

## 4 Discussion

In this study we explored the behavioural consequences of a lesion of the caudal right pre-SMA on three standard measures of cognitive control. Our aim was to identify whether KP's behaviour had changed as a result of the lesion and how this could be integrated into contemporary accounts of pre-SMA function. To our knowledge the lesion described here is unique in the literature as it does not extend into neighbouring anterior cingulate cortex or SMA.

We employed tasks designed to index specific aspects of executive function or cognitive control in order to stratify the behavioural effects of the lesion. We explored whether responses that require inhibition of pre-potent response (STOP task), updating of a response plan (CHANGE task), or inhibition of distractors (Eriksen flanker) were affected when performance was compared to a control group. We found that KP demonstrated a specific deficit when rapidly updating a response plan as assessed by the CHANGE task. However, no significant deficits were observed when KP was required to withhold a response on the STOP task or during situations where conflict occurred at the level of the stimulus, as in the Eriksen flanker task (except generalised slowing).

**Fig. 4:**
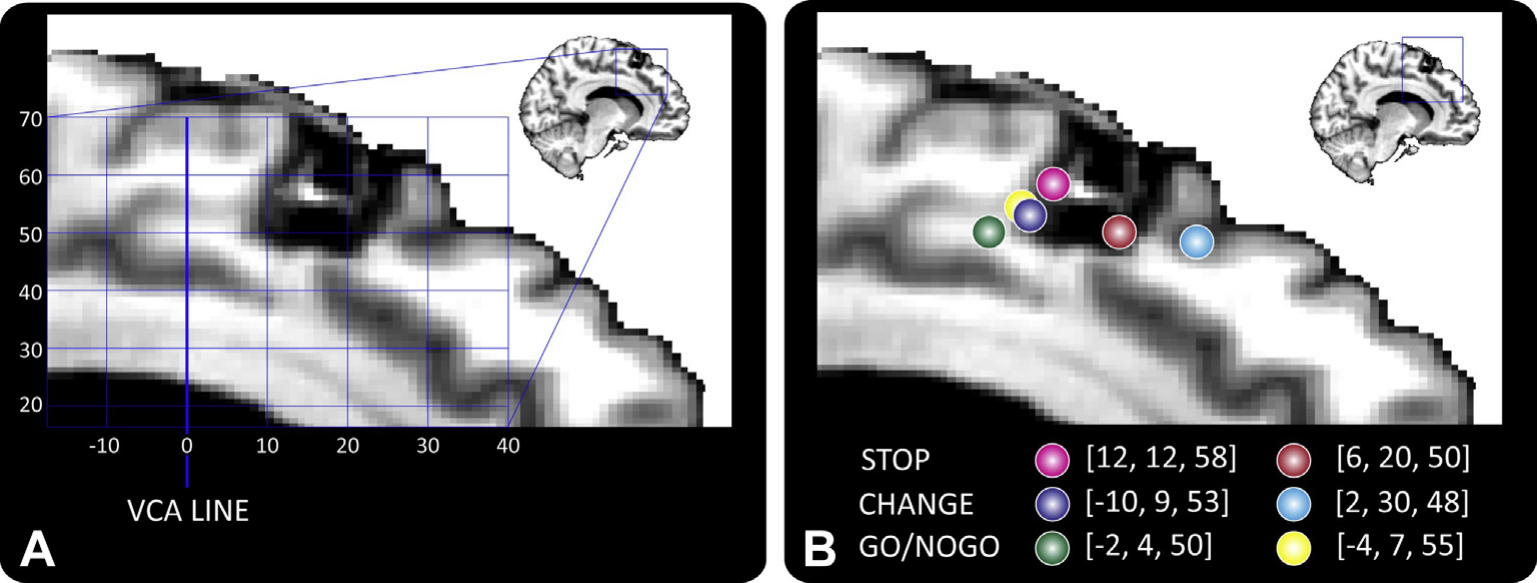
Lesion location with respect to previous functional imaging studies. The location of functional activations in pre-SMA from six previous studies which employed cognitive control tasks are superimposed on the structural image of the lesion location in MNI space to illustrate the proximity of the lesion to previous functional imaging results. The studies include STOP-signal (Aron & Poldrack, 2006; Sharp et al., 2010), Go-NoGo (Garavan et al., 2003; Mostofsky et al., 2003) or designs where the response plan had to be rapidly updated or changed (Nachev et al., 2005; Rushworth et al., 2002). All the studies have >100 citations and were chosen for illustrative purposes rather than as a comprehensive meta-analysis. The axial and sagittal coordinates were used in order to demonstrate the rostro-caudal extend of the lesion with respect to previous findings.

The location of the lesion with respect to medial frontal activations from several previous experiments which were designed to isolate brain responses associated with either stopping or changing a response plan is shown in Fig. 4A and B. There is clearly a high degree of overlap with activation foci from tasks requiring either stopping or changing a response plan, yet in this patient we only observed a deficit in action updating. This illustrates the challenge for interpretation of these behavioural findings. We now attempt to place this finding in the context of current theories of medial frontal cortical function.

One approach to explaining the relationship between brain function and cognitive control is to examine the complexity of the response required for a given task. Classifying paradigms with respect to their complexity potentially provides a single metric to distinguish different tasks (Nachev et al., 2008), and offers a way to interpret the range of behaviour which has been associated with the pre-SMA (Behrens et al., 2012). For example, performance on the STOP task requires an on-going response to be inhibited, whereas the CHANGE task might first require inhibition of the prepared response and *then* execution of the alternate response. As the CHANGE task is computationally more complex than the STOP task, these tasks might recruit different brain areas.

It has been suggested that such differences in functional complexity could be encoded along a rostro-caudal gradient within the supplementary motor complex (SMC), an area which includes both pre-SMA and SMA (Nachev et al., 2008). In this model, more rostral areas are associated with a higher degree of conflict processing or complexity of response than caudal regions. What evidence is there that such a gradient exists in SMC?

Neuroimaging and lesion evidence in humans, and neurophysiology in monkeys suggests that increasingly complex tasks are more often associated with rostral SMC areas (Matsuzaka & Tanji, 1996; Nachev et al., 2005; Picard & Strick, 1996; Picard & Strick, 2001), with caudal regions more strongly associated with action execution, but it remains unclear the degree of functional granularity it is possible to detect within this region. In terms of brain structure, pre-SMA and SMA are separable based on their patterns of structural connectivity in both humans and monkeys (Inase, Tokuno, Nambu, Akazawa, & Takada, 1999; Johansen-Berg et al., 2004). Furthermore, in humans, pre-SMA has been parcellated into anterior and posterior regions based on differences in functional connectivity (Zhang, Ide, & Chiang-shan, 2012). As the resolution of these techniques improves, further sub-divisions may also be detectable.

In the context of the lesion described here, the complexity model predicts that stopping responses could be initiated by structures other than pre-SMA. One possible candidate is adjacent, caudally located SMA, where stimulation or lesions have been found to affect the ability to inhibit actions (Drewe, 1975; Fried et al., 1991; Picton et al., 2007), and which has also been associated with automatic, unconscious inhibition of voluntary actions (Sumner et al., 2007). Therefore it might be possible that pre-SMA is not specifically required for stopping, and instead plays a more important role in switching response plans.

A challenge to this interpretation comes from recent work where pre-SMA activity was modulated using TMS during performance of a response inhibition task. The authors reported that implementation of the stopping process was disrupted *without* affecting the ability to update response plans (Cai et al., 2012; Obeso et al., 2013). Macrostimulation of pre-SMA in humans has also been found to halt motor responses (Filevich, Kühn, & Haggard, 2012; Swann et al., 2012). Although these studies suggest that pre-SMA is directly involved in stopping responses, the use of SMA as a control site could have extended their findings, and the possibility of non-localised effects of the stimulation modalities cannot be entirely discounted, particularly since SMA is directly adjacent to pre-SMA. However, if stimulation of pre-SMA can inhibit a response but a lesion of the caudal pre-SMA does *not* affect stopping, how can these apparently inconsistent positions be reconciled?

One approach is to consider whether inhibitory control of behaviour might not be governed by a unitary system. In humans, although the Go-NoGo and STOP-signal paradigms have often been grouped collectively under the term ‘response inhibition’, they are actually associated with quantitatively different patterns of activation (Swick, Ashley, & Turken, 2011) – suggesting that ‘not going’ and ‘stopping’ are not necessarily synonymous. Recently it has been proposed that inhibiting a response might be achieved in two different ways: reactive and proactive (Aron, 2011). *Reactive* inhibition is conceptualised as a *global* stopping mechanism analogous to the handbrake in a car, whereas *proactive* inhibition is a *selective* system deployed when stopping is anticipated, more like a footbrake.

The neuroanatomical evidence for the existence of separate response inhibition pathways comes from monkey neurophysiology studies. Here pre-SMA and SMA have been found to maintain separate projections with two subcortical regions that have frequently been associated with response inhibition: the STN and striatum (Inase et al., 1999). The frontosubthalamic and frontostriatal pathways are thought to mediate ‘hyperdirect/reactive’ and ‘indirect/proactive’ modes of inhibition respectively. Evidence from intracellular recordings suggests that the convergence of these pathways in the basal ganglia may explain their complementary functionality. When STN and globus pallidus neurons are activated in response to cortical or corticofugal stimulation, they are subsequently inhibited via activation of the slower frontostriatal projection (Smith, Beyan, Shink, & Bolam, 1998). Although the microcircuitry of the basal ganglia is highly complex and still not fully understood, this feedback mechanism might facilitate the process of halting an action in order to then initiate an alternative response, and provides a possible explanation for the existence of separate cortico-subcortical inhibitory pathways.

In humans, changes in motor-evoked potentials (MEPs) recorded during performance of response inhibition tasks have been used to explore how differences in task requirements can affect the rest of the motor system. In a simple STOP-signal task that required only a left or right thumb press in response to the direction of a go signal, suppression of motor activity in successful STOP trials was observed bilaterally in both hand *and* leg muscles up to 400 msec after the stimulus was presented (Badry et al., 2009). Thus this result appears to exemplify *global* inhibition. In a separate experiment where participants were cued as to which hand movement they were likely to have to inhibit, preparatory suppression was observed more *specifically*, occurring only in the cued effector muscles (Claffey, Sheldon, Stinear, Verbruggen, & Aron, 2010). These findings suggest that inhibition can be applied globally or in a selective fashion depending on the behavioural context. They may therefore reflect the difference between deployment of reactive vs. proactive inhibition.

If there are different mechanisms for inhibition, how could this explain the findings reported here in our patient? Consider a situation where reactive inhibition is initiated by SMA and proactive inhibition by pre-SMA. First, following a lesion of the pre-SMA region mediating proactive inhibition, performance of the STOP task would remain intact if reactive stopping were mediated by SMA. Paradoxically, response times might even improve, as it would minimize involvement of the slower, frontostriatal selective stopping mechanisms. Second, in a situation where there is a selective deficit in proactive inhibition, performance of the CHANGE task would now have to rely on the reactive inhibitory mechanisms. Thus instead of being able to *selectively* halt a pre-potent response and update it, the reactive global stopping response would have to be deployed.

Deploying such a mechanism might be possible but comes at a cost. STN stimulation in Parkinson's disease – which may affect the hyperdirect/reactive pathway – improves performance on STOP and Go-NoGo tasks (Van den Wildenberg et al., 2006), but also results in cortical inhibition-related activity which persists for up to 400 msec (Baker, Montgomery, Rezai, Burgess, & Lüders, 2002). Suppression of motor output over a similar timescale due to global inhibition has also been observed using MEPs (Badry et al., 2009). These data suggest that although the CHANGE task could be performed using the reactive inhibitory pathway, this would come at the cost of a delay due to the duration of the post-stimulus suppression. Thus, caudal pre-SMA may not be necessary for stopping *per se*, but might be more important for *selectively inhibiting* an action plan in order to switch to an alternative response. This possibility is supported by evidence from studies of neurons in monkey pre-SMA and functional imaging in humans which suggest that pre-SMA may be crucial for switching between controlled and automatic behaviour (Forstmann et al., 2008; Isoda & Hikosaka, 2007). Thus, it is likely that this patient might also exhibit elongated reaction times on tasks which specifically test the ability to switch between response plans. Unfortunately, we did not have the opportunity to test this.

As there is evidence to suggest that focal lesions can also result in disruption of network activity (Gratton et al., 2012), and since pre-SMA is thought to form a part of a right-lateralised inhibitory network (Aron et al., 2007), to what extent can it be reasonably argued that these findings are attributable to deficits *solely* in pre-SMA function? First, the lesion is a consequence of a resection, rather than vascular pathology, and is highly constrained within the grey matter, therefore it is unlikely that the observed behaviour is the result of a pure disconnection syndrome. Second, this distinct deficit in switching between responses is consistent with previous electrophysiological recordings in monkey pre-SMA (Isoda & Hikosaka, 2007, 2008), whereas the function of the other regions involved in this inhibitory network, IFC and STN, has been more consistently associated with either stopping responses or attentional capture (Aron & Poldrack, 2006; Sharp et al., 2010; Swann et al., 2012), behaviours in which we observed no deficit at all. However, future studies may still wish to consider employing functional or structural neuroimaging – such as DTI or resting state – in order to test for possible differences in *network* function following such lesions.

The lateralisation of the lesion to the right hemisphere raises the question of whether a patient presenting with a left hemisphere lesion would demonstrate a similar deficit. The extant evidence places right pre-SMA as a node in a right-hemisphere network involved in response inhibition (Aron et al., 2007), but to our knowledge no similar network has been identified in the left hemisphere. A recent meta-analysis suggests that right pre-SMA is more strongly activated in response to increased task difficulty – situations which are very likely to involve an element of selection or response switching (Keuken et al., 2014). Therefore it appears that there is evidence to suggest that left and right pre-SMA may perform different functions, but how much these reflect hemispheric specialisations and differences in task design remains an open question.

This discussion has focused on the role of pre-SMA and SMA in stopping and switching response plans. Other regions within medial frontal cortex, particularly ACC, have also been implicated in stopping responses (Botvinick et al., 1999). Lesion studies have demonstrated functional heterogeneity within ACC, with the behavioural deficits dependent on the modality of response (Turken & Swick, 1999), and more often associated with deficits in error detection and correction (Ullsperger & von Cramon, 2006). The Eriksen Flanker differs fundamentally from the STOP and CHANGE paradigms because it activates conflicting responses simultaneously, analogous to the Stroop effect, rather than via two separate stimuli presented at different temporal intervals. This may explain why we did not observe any significant behavioural deficits on this paradigm, except generalised slowing. These data might arguably be considered to be consistent with the proposal that ACC does not activate when only stimulus selection is required, but instead appears to provide an evaluative and error monitoring function in situations of conflict (Rushworth, Walton, Kennerley, & Bannerman, 2004; Swick & Turken, 2002).

In conclusion, our finding of a dissociation between stopping and switching actions following a lesion of caudal pre-SMA sheds new light on the role of this brain area in the control of action. The results suggest that caudal pre-SMA plays an important role in facilitating selective inhibition, either by promoting this directly or by initiating transitions between reactive and proactive inhibitory mechanisms. Future investigations might profitably consider the distinction between reactive and proactive mechanisms when developing tasks to probe the fundamental function of pre-SMA.

## Figures and Tables

**Table 1:** Testing protocol for patient KP. A) Neuropsychological test battery for intellectual functioning. B) Focal tests of cognitive function. C) Testing protocol for the experiments described in this chapter. KP was tested on the CHANGE, STOP and Flanker tasks over a 10 week period. The numbering 1–3 indicates in which session the data was acquired.

A)			
Intellectual functioning	69 days prior	14 days prior	106 days post
Verbal IQ (WAIS-III)	98	Not tested	98
Performance IQ (WAIS-II)	111	Not tested	125
Advanced Progressive Matrices	Not tested	41st %ile	56th %ile
**Memory**			
Recognition Memory Test Words	50th %ile	25–50th %ile	50th %ile
Recognition Memory Test Faces	At chance	50–75th %ile	90th %ile
Doors and People – People Test	Not tested	50th %ile	50–75th %ile
Doors and People – Shapes Test	Not tested	75 %ile	75th %ile

B)		
Focal cognitive	Prior	Post
Naming Skills	50–75th %ile	95th %ile
VOSP Silhouettes and Cube Analysis	>5% cut off	>5% cut off
Stroop colour-word	Very superior	Very superior
Trails B	90th %ile	75–90 %ile
Symbol Digit Modalities Test	average	mild impaired

**C)**			
Days post-surgery	31	71	104
Session	1	2	3
**Task**			
CHANGE task	X	X	X
STOP task		X	
Eriksen Flanker		X	X
